# The value of [68Ga]Ga-DOTA-TATE PET/CT in diagnosis and management of suspected pituitary tumors

**DOI:** 10.1186/s41824-021-00104-3

**Published:** 2021-05-24

**Authors:** Fuad Novruzov, Aziz Aliyev, Ming Young S. Wan, Rizwan Syed, Elnur Mehdi, Irada Aliyeva, Francesco Giammarile, Jamshed B. Bomanji, Irfan Kayani

**Affiliations:** 1 Department of Nuclear Medicine, Azerbaijan National Centre of Oncology, M. Xiyabani street 137, AZ 1011 Baku, Azerbaijan; 2 Institute of Nuclear Medicine, University College London Hospital, London, UK; 3 Department of Head and Neck Surgery, Azerbaijan National Centre of Oncology, Baku, Azerbaijan; 4 Department of Internal Medicine, Azerbaijan Medical University, Baku, Azerbaijan; 5 International Atomic Energy Agency, Vienna, Austria

## Abstract

**Background:**

Gallium 68-tetraazacyclododecane-tetraacetic acid-octreotate ([68Ga]Ga-DOTA-TATE) is a selective somatostatin analogue ligand, which shows increased affinity for somatostatin receptor subtype (SSTR) 2 and has been used routinely for imaging neuroendocrine tumors with PET/CT. We investigated the utility of [68Ga]Ga-DOTA-TATE positron emission tomography/computed tomography (PET/CT) in patients with suspected pituitary pathology. We reviewed imaging for twenty consecutive patients (8 men, 12 women, mean age of 48.2, range 14–78) with suspected pituitary pathology who were referred for [68Ga]Ga-DOTA-TATE PET/CT.

**Results:**

Nine patients presented with recurrent Cushing’s syndrome following surgical resection of pituitary adenomas due to recurrent Cushing’s disease (seven patients) and ectopic ACTH secreting tumor (2 patients). All seven patients with recurrent Cushing’s disease showed positive pituitary [68Ga]Ga-DOTA-TATE uptake while both cases of ectopic hormonal secretion had absented pituitary uptake. In 1 of these 2 patients, [68Ga]Ga-DOTA-TATE was able to localize the source of ectopic ACTH tumor.

Six patients presented de novo with Cushing’s due to ectopic ACTH secretion; [68Ga]Ga-DOTA-TATE PET/CT was able to localize ectopic tumors in six of eight patients (3 lungs, 2 pancreases, 1 mid-gut)

There was high uptake [68Ga]Ga-DOTA-TATE in 3 cases of recurrent central hyperthyroidism (SUVmax 6.6–14.3) and 2 cases of prolactinoma (SUVmax 5.5 and 11.3).

**Conclusion:**

Absent [68Ga]Ga-DOTA-TATE activity in the pituitary fossa is useful in excluding pituitary disease in recurrent Cushing’s. Recurrent pituitary thyrotropinomas and prolactinomas showed moderate to high pituitary activity. In addition, in Cushing’s syndrome, [68Ga]Ga-DOTA-TATE is useful for detection of ectopic sources of ACTH production, especially where anatomic imaging is negative.

## Introduction

Neuroendocrine tumors (NET) cover a heterogeneous group of tumors, which originate from endocrine glands (pituitary, parathyroid, adrenal medulla) or other endocrine organs like thyroid, pancreas, respiratory, and gastrointestinal tissue.

As most NETs express somatostatin receptors, they can be adequately targeted and visualized with somatostatin receptor radio-labeled analogs in vivo (Guyton and Hall 2000; Newell-Price et al. 2006; Gadelha and Vieira 2014; Bombardieri et al. 2001).

The use of Gallium 68-tetraazacyclododecane-tetraacetic acid-octreotate ([68Ga]Ga-DOTA-TATE labeled for the somatostatin receptor scintigraphy (SRS) is based on the increased affinity of [68Ga]Ga-DOTA-TATE labeled somatostatin receptor ligands relative to ^111^In-octreotide (Newell-Price et al. 2006; Gadelha and Vieira 2014; Bombardieri et al. 2001; Balon et al. 2001; Bombardieri et al. 2010). European Neuroendocrine Tumor Society guidelines (Balon et al. 2001; Haug et al. 2010) recommend the use of PET/CT for the localization of the primary tumor in metastatic NETs (Kwekkeboom et al. 2009).

The aim of our study was to evaluate the utility of [68Ga]Ga-DOTA-TATE PET/CT imaging scan in patients with suspected pituitary pathology. Patients were divided into two broad groups: those with ACTH dependent Cushing’s syndrome and those with recurrent prolactinomas and thyrotropinomas.

Cushing’s syndrome is a hormonal imbalance due to abnormally increased levels of cortisol hormone in blood. Cushing’s syndrome is divided into 2 types: ACTH-dependent and ACTH-independent forms. In ACTH-dependent type, there is over-synthesis of ACTH from pituitary adenoma, called Cushing’s disease (CD), or ectopic secretion of ACTH from peripheral tumors (Antunes et al. 2007). CD is the most common form of endogenous Cushing’s syndrome, accounting for approximately 70% of cases (Pape et al. 2012; Prasad et al. 2010). Ectopic ACTH secretion is a cause of approximately 15–20% of ACTH-dependent Cushing’s syndrome (Tabarin et al. 1999). In the literature, several small case series studies have reported on use of [68Ga]Ga-peptide ligands to evaluate ectopic ACTH secreting tumors (Tsagarakis et al. 2003; Veit et al. 2013; Singer et al. 2010; Haug et al. 2012). Prolactinomas are relatively common primary pituitary neoplasms whereas thyrotropinomas are rare. In both cases, however, there is very limited literature on use of somatostatin receptor imaging in vivo.

## Methods

### Patients

A search of our Institutional database over 5-year period between 2008 and 2013 revealed 20 consecutive patients (8 male, 12 females with mean age 48.2 years (range 14–78 years)) who underwent [68Ga]Ga-DOTA-TATE PET/CT for evaluation of pituitary pathology.

The indication for [68Ga]Ga-DOTA-TATE PET/CT were as follows (Table 1):

- Suspected recurrent Cushing’s disease following previous surgical resection
- ACTH dependent Cushing syndrome secondary to suspected ectopic ACTH production
- Recurrent central hyperthyroidism
- Recurrent prolactinoma

**Table 1:** Patients’ demographic and clinical characteristic

Total number of enrolled patients	20
**Gender of patients**	
Male	**8**
Female	**12**
**Clinical presentation**	
**Suspicious recurrent Cushing disease**	**9**
**ACTH-dependent ectopic Cushing syndrome**	**6**
**Recurrent central hyperthyroidism**	**3**
Recurrent galactorrhea	**2**

### PET/CT acquisition parameters

Images were acquired 45–60 min after injection of 120–200 MBq of [68Ga]Ga-DOTA-TATE. Imaging was performed using a dedicated GE Discovery STE camera combining a PET unit and a 16-slice CT unit; whole-body examinations (brain to mid-thigh) were performed with the patient supine. The CT exposure factors for all examinations were 120 kVp and 80 mA in 0.8 s. Maintaining patient position, we performed a whole-body PET emission scan covering an area identical to that covered by CT. PET scans were acquired at a rate of 4 min per bed position, and PET images were reconstructed using CT for attenuation correction. The [68Ga]Ga-DOTA-TATE PET acquisitions were performed in 3 dimensions with a 5-slice overlap between consecutive bed positions. Ga-68-DOTATATE PET images were reconstructed using an ordered-subsets expectation maximization algorithm with 3 iterations and 25 subsets. The CT data for [68Ga]Ga-DOTA-TATE were reconstructed to axial slices 3.75 mm thick with a soft-tissue reconstruction algorithm and 2.5 mm thick with a lung reconstruction algorithm.

### Image analysis

The documented clinical reports were used to determine results of [68Ga]Ga-DOTA-TATE PET/CT scans. The presence or absence of uptake at the suspected lesion level allowed to classify the 20 patients in “positive” and “negative” respectively. In addition, scans were retrospectively reviewed to document standardized uptake value (SUVmax) in all lesions.

Histological confirmation of tumor type was available for all patients except for one case where ectopic ACTH source for Cushing syndrome was unknown.

All patients had informed consent, and institutional board ethics approval was received for this retrospective study.

## Results

Tumor overview, histology assessment, and [68Ga]Ga-DOTA-TATE uptake are summarized in Table 2.

**Table 2:** Summary of tumor characteristic and finding

***Patients No***	***Age, sex***	***Referral presentation***	***Previous treatment (PS/RT)***	***Tumor site/***^***68***^***Ga-PET/CT uptake***	***Final diagnosis/histology***
1	68, M	Suspected recurrent Cushing disease	PS	Pituitary fossa (SUVmax 2.3)	CD/pituitary adenoma with expression of ACTH
2	37, M	Suspected recurrent Cushing disease	PS	Pituitary fossa (SUVmax 4.2	CD/pituitary adenoma with expression of ACTH
3	57, F	Suspected recurrent Cushing disease	PS	Pituitary fossa (SUVmax 4.2)	CD/atypically pituitary adenoma with expression of ACTH
4	49, F	Suspected recurrent Cushing disease	PS	Pituitary fossa (SUVmax 3.1)	Pituitary adenocarcinoma with expression of ACTH
5	26, M	Suspected recurrent Cushing disease	PS	Pituitary fossa (SUVmax 6.1)	CD/pituitary adenoma with expression of ACTH
6	78, F	Suspected recurrent Cushing disease	PS + RT	Pancreas (SUVmax 8.4)	Pancreatic NET’s with expression of ACTH
7	30, F	Suspected recurrent Cushing disease, central hyperthyroidism	PS	Pituitary fossa (SUVmax 5.2)	CD+ TSH-oma/plurihormonal pituitary adenoma with expression of TSH, FSH, and ACTH
8	68, F	Suspected recurrent Cushing disease	PS	Pituitary fossa (SUVmax 3.9)	Cyclical CD/adenoma with expression of ACTH
9	67, F	Suspected recurrent Cushing disease	PS	Negative	Ectopic ACTH secreting tumor, unknown source
10	73, F	Ectopic Cushing syndrome	NO	Negative	Pancreatic NET’s with expression of ACTH
11	14, M	Ectopic Cushing syndrome	NO	Lung nodule (SUVmax1.4)	Atypical lung carcinoid with expression ACTH
12	58, M	Ectopic Cushing syndrome	NO	Lung nodule (SUVmax 1.9)	Typical lung carcinoid with expression of ACTH
13	22, F	Ectopic Cushing syndrome	NO	Lung nodule (SUVmax 2.0)	Typical lung carcinoid with expression of ACTH
14	42, F	Ectopic Cushing syndrome	NO	Small bowel (SUVmax 25.3)	Metastatic mid-gut NET’s with expression of ACTH
15	49, F	Ectopic Cushing syndrome	NO	Head of pancreas (SUVmax 35.5	Pancreatic NET’s with expression of ACTH
16	27, M	Recurrent central hyperthyroidism	PS	Pituitary fossa (SUVmax 6.6)	TSH-Oma/pituitary adenoma with expressing of TSH
17	48, M	Recurrent central hyperthyroidism	PS	Pituitary fossa (SUVmax 6.7)	TSH-Oma/pituitary adenoma with expressing of TSH
18	50, F	Recurrent central hyperthyroidism	PS	Pituitary fossa (SUVmax 14.3)	TSH-Oma/pituitary adenoma with expressing of TSH
19	23, M	Pituitary mass	EBRT	Pituitary fossa (SUVmax 5.5)	Prolactinoma/pituitary adenoma with expression of prolactin
20	34, F	Recurrent galactorrhea	NO	Pituitary fossa (SUVmax 11.3)	Prolactinoma/pituitary adenoma with expression of prolactin

*PS* pituitary surgery, *EBRT* external beam radiation therapy, *CD* Cushing’s disease, *ACTH* adrenocorticotropic hormone, *TSH* thyroid stimulating hormone, *NET* neuroendocrine tumor

Fifteen patients had Cushing’s syndrome. Of these 15, nine presented with recurrent Cushing’s following surgical treatment for Cushing’s disease. Six out of 15 patients presented de novo with ectopic ACTH-dependent Cushing’s syndrome. In 7/9 patients with recurrent Cushing’s syndrome, there was recurrent pituitary disease. In 2/9 patients, recurrent Cushing’s syndrome was due to ectopic ACTH producing tumor.

The source of ectopic ACTH was due to bronchial carcinoid (3 patients), pancreatic NETs (2 patients), and mid gut NET (1 patient). Of 3 bronchial carcinoid tumors, 2 were typical carcinoid (0.8 and 1.7 cm) and 1 was atypical carcinoid (1.5 cm). In one patient, ectopic source of ACTH production was unknown.

In all seven patients with recurrent Cushing’s secondary to recurrent Cushing’s disease, there was positive uptake of [68Ga]Ga-DOTA-TATE within pituitary (SUVmax 2.3–6.1, mean 4.1). In both cases of recurrent Cushing’s due to ectopic ACTH production, there was absent uptake of [68Ga]Ga-DOTA-TATE in the pituitary. Pituitary uptake in those with recurrent pituitary adenomas was less intense than pituitary uptake seen in patients presenting de novo with ectopic Cushing’s (SUVmax 4.8–8.9, mean 6.2).

**Fig. 1:**
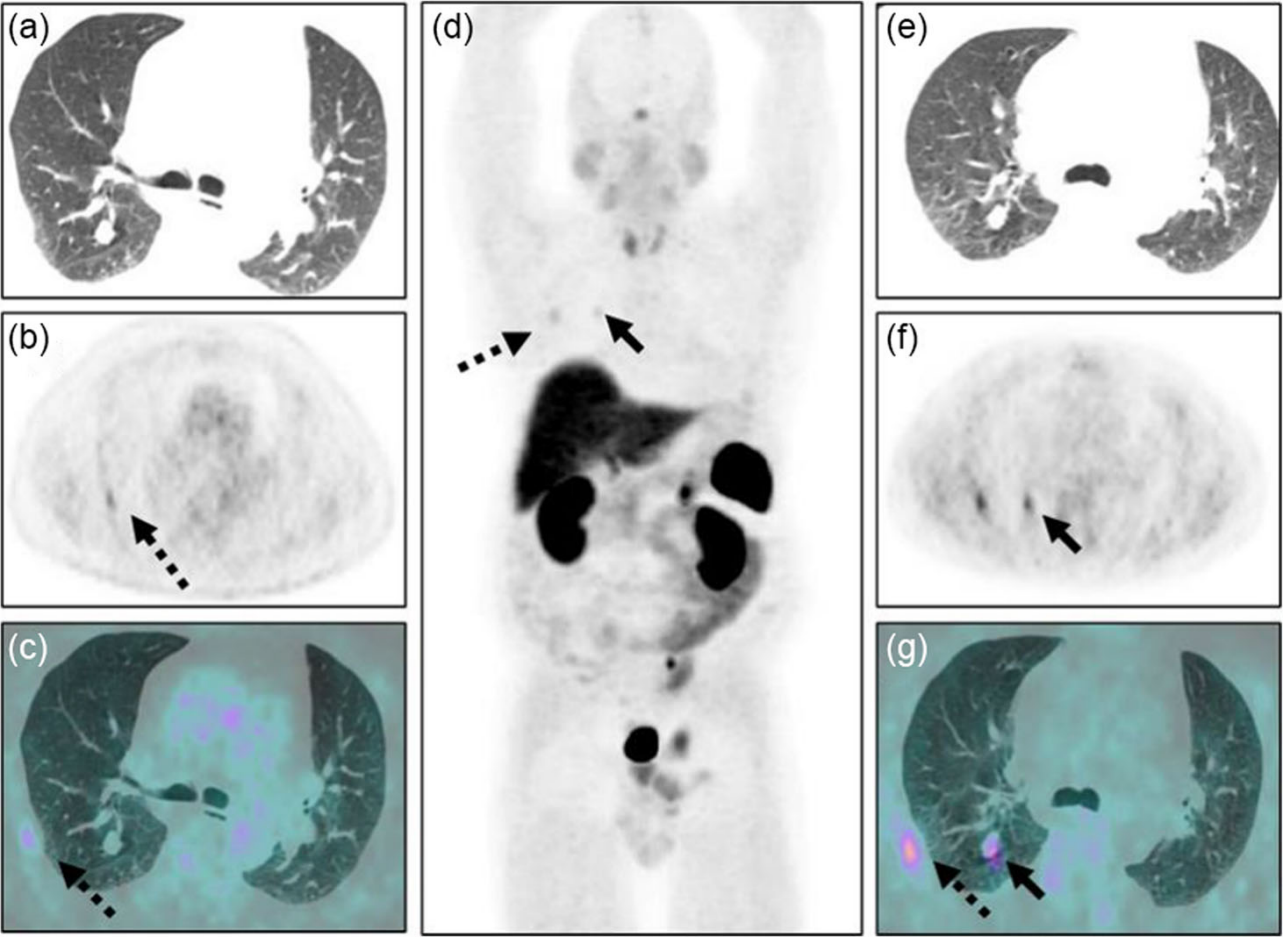
A 58-year-old man was presented with ectopic ACTH secreting Cushing syndrome. FDG PET/CT images (**a**–**c**) showed a non-avid lung nodule. MIP, trans-axial CT, PET, and fused PET/CT images (**d**–**g**) show mild focal [68Ga]Ga-DOTA-TATE uptake (SUVmax 1.9) in right lower lobe nodule (black filled arrows). Post-surgery histological diagnosis was typical bronchial carcinoid secreting ACTH. After surgery Cushing’s symptoms is cured. Uptake is also seen with FDG (**b**, **c**) and [68Ga]Ga-DOTA-TATE (**d**, **f**, **g**) PET/CT due to a rib fracture (black dashed arrows)

**Fig. 2:**
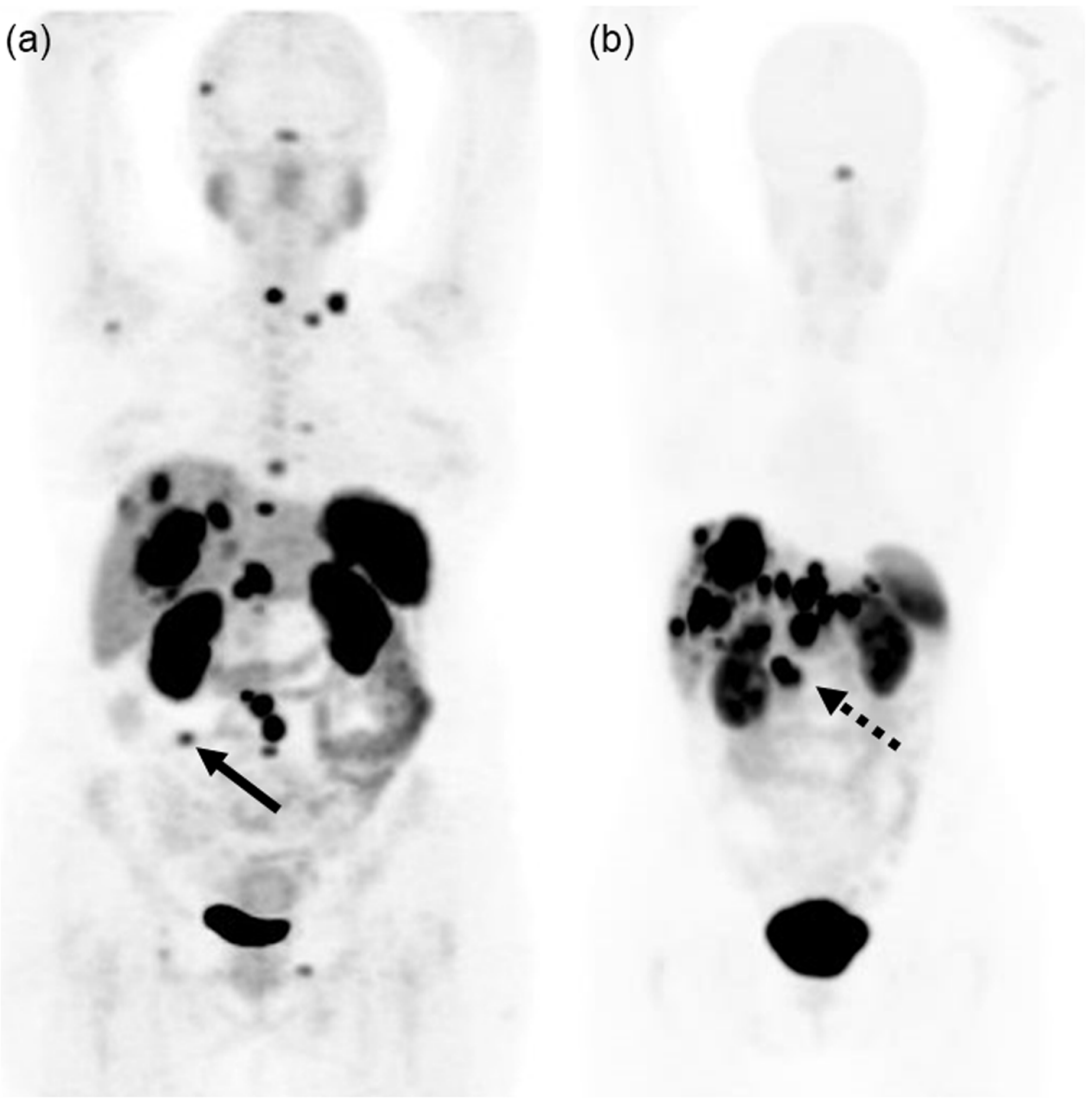
A 2 [68Ga]Ga-DOTA-TATE PET MIP (**a**, **b**) images in two female patients, 42-year and 49-year-old, with ectopic ACTH secreting Cushing syndrome due to metastatic neuroendocrine tumors. Primary tumor sites in both patients were identified by [68Ga]Ga-DOTA-TATE as shown by focal intense uptake (SUVmax 25.3 and 35.5) at small bowel (filled arrow) and head of pancreas (dashed arrow). Both patients subsequently underwent for somatostatin receptor radionuclide therapy

[68Ga]Ga-DOTA-TATE was able to depict source of ectopic ACTH production in six of eight patients (1/2 patients with recurrent Cushing’s syndrome, and 5/6 patients presenting de novo). [68Ga]Ga-DOTA-TATE showed positive but low uptake (Fig. 1) in all three bronchial carcinoids (SUVmax 1.4–2.0). There was high [68Ga]Ga-DOTA-TATE uptake in 1/2 Pancreatic NETs (SUVmax 35.5) and one Mid-Gut NET (SUVmax 25.3) (Fig. 2).

**Fig. 3:**
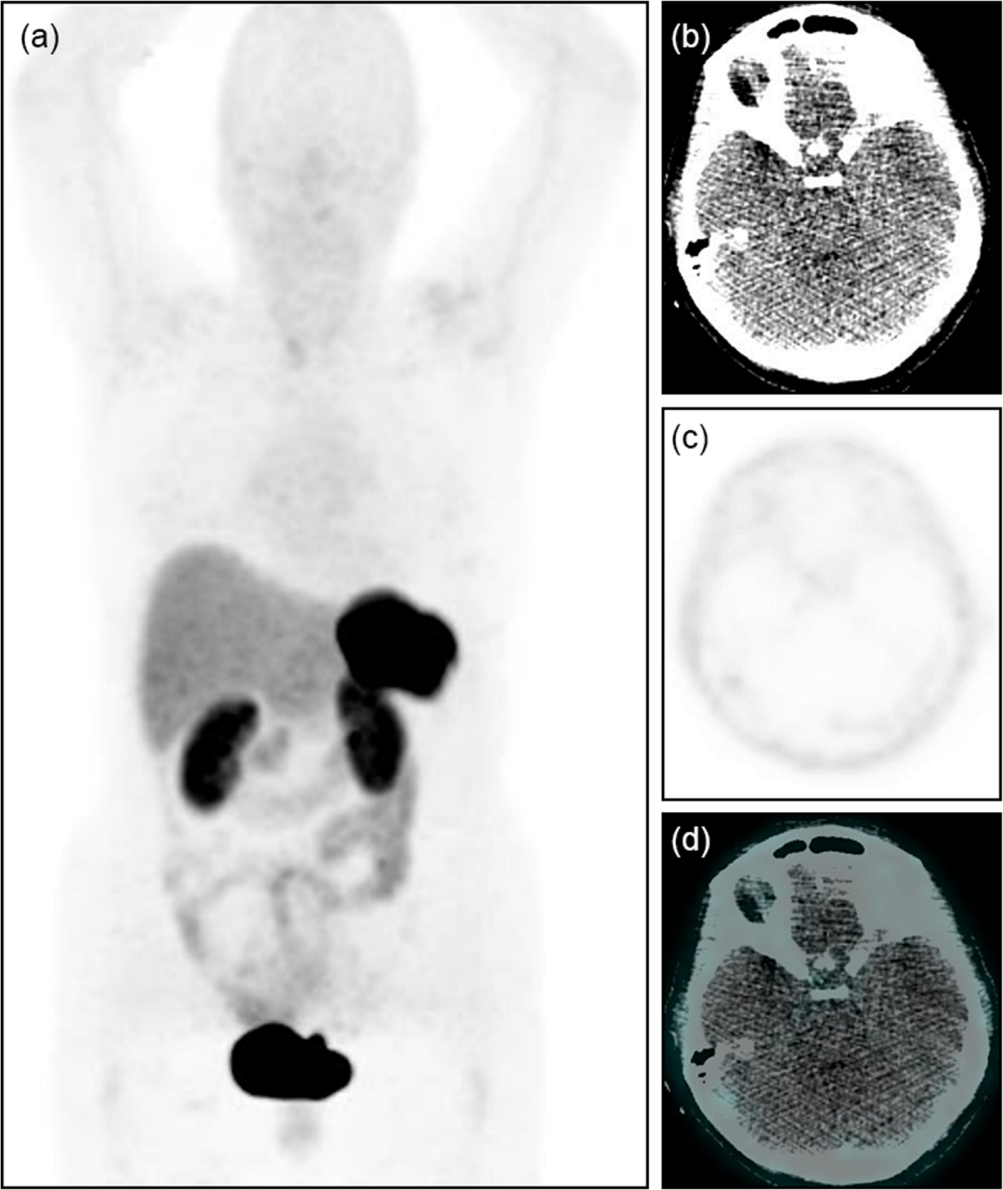
A 67-year-old woman history of surgical resection for ACTH pituitary macro-adenoma was referred with suspected recurrent Cushing disease. PET MIP (**a**), trans-axial CT, PET, and fused PET/CT images (**b**–**d**) show no [68Ga]Ga-DOTA-TATE uptake in pituitary fossa in keeping with prior curative resection. MRI showed no glandular tissue in pituitary fossa. The source of recurrent ACTH in this case was unknown

In one with negative [68Ga]Ga-DOTA-TATE uptake, ectopic source of tumor was not shown on any imaging modality, ectopic tumor ACTH production was diagnosed biochemically and on basis of complete resection of pituitary tissue (shown on MRI) as well absent pituitary [68Ga]Ga-DOTA-TATE activity (Fig. 3). In another with pancreatic NET, there was negative [68Ga]Ga-DOTA-TATE uptake as well as negative CT and MRI with tumor depicted only on EUS.

In one case with unknown primary site on conventional CT/MRI imaging Ga-[68Ga]Ga-DOTA-TATE showed site of primary tumor (in terminal ileum).

**Fig. 4:**
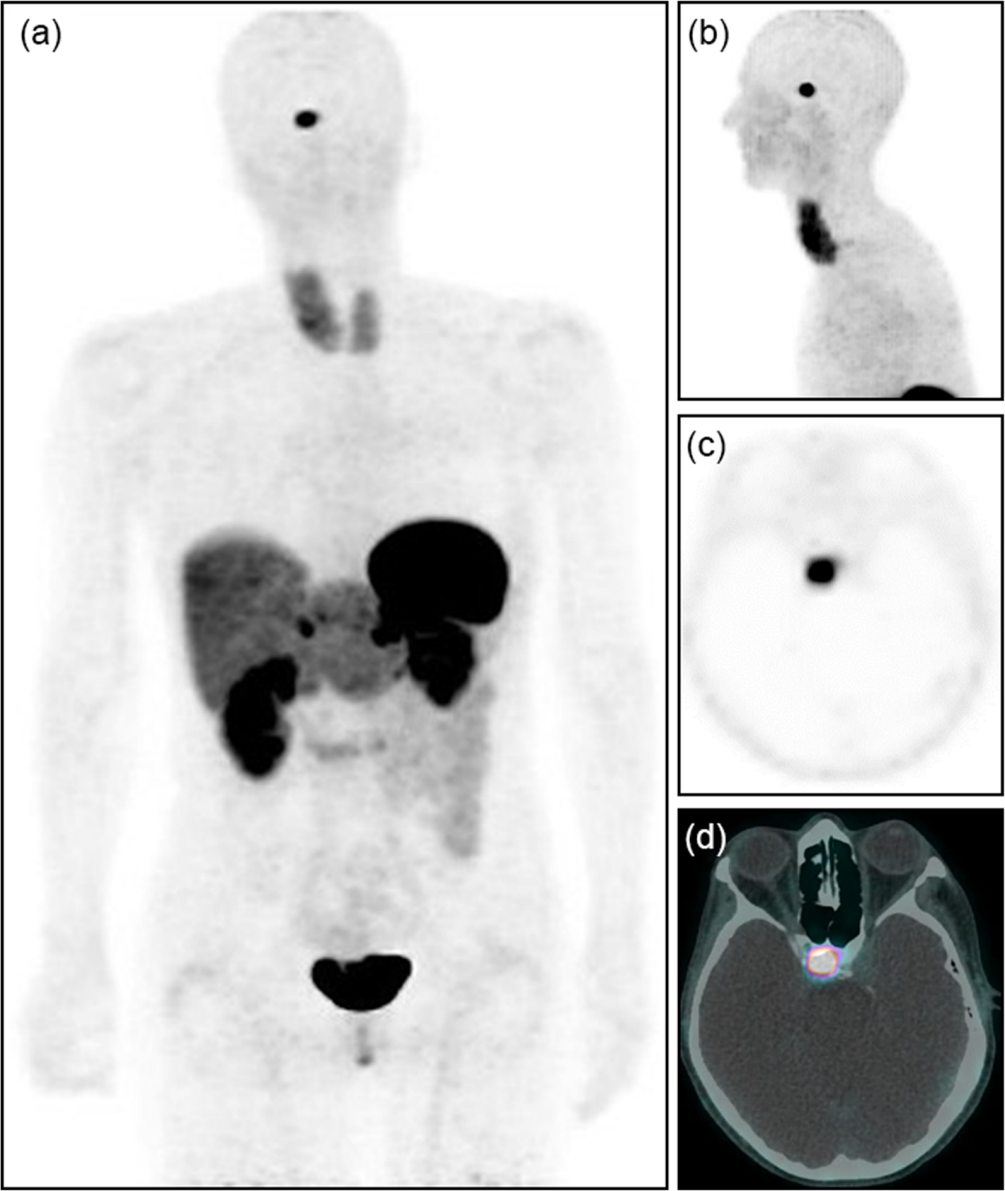
A 50-year-old woman was referred with recurrent central hyperthyroidism, 2 year post pituitary surgery for a TSH secreting micro-adenoma. [68Ga]Ga-DOTA-TATE PET MIP (**a**, **b**), trans-axial PET, and fused PET/CT images (**c**, **d**) clearly depict increased focal uptake (SUVmax 14.3) in pituitary fossa. The patient underwent repeat surgical resection of recurrent pituitary adenoma (Thyrotropinoma)

Three patients presented with recurrent central hyperthyroidism due to thyroid stimulating hormone (TSH) secreting adenoma following previous surgical resection, with increased TSH and free-thyroid hormone levels, and residual pituitary macro adenomas on MRI (size 13 mm, range 11–15 mm). All patients with recurrent thyrotropinomas showed high tracer uptake (Fig. 4) within pituitary (mean SUVmax 9.2, range 6.7–14.3). Two patients with pituitary adenoma secondary to prolactinoma showed moderate to high [68Ga]Ga-DOTA-TATE uptake in pituitary gland (SUVmax 5.5 and 11.3).

## Discussion

Our study suggests that, in selected indications, [68Ga]Ga-DOTA-TATE has a useful role in evaluating patients with suspected pituitary pathology.

[68Ga]Ga-DOTA-TATE activity within the pituitary fossa is a marker for functioning pituitary tissue, a property which can help assess patients with recurrent Cushing’s syndrome following resection of corticotrophin secreting pituitary tumors. Positive pituitary uptake indicates the presence of functioning pituitary tissue; in all seven patients with recurrent Cushing’s disease, there was positive uptake within pituitary although this was less than normal pituitary activity seen in those with Cushing’s due to ectopic ACTH secretion. Our findings are in keeping with Zhao et al. who showed that [68Ga]Ga-DOTA-TATE had higher uptake in normal remaining pituitary tissue than in recurrent or residual pituitary adenomas (Invitti et al. 1999). In contrast, both patients with recurrent Cushing’s syndrome due to ectopic ACTH secretion had no uptake within pituitary, in keeping with treated pituitary disease.

The diagnosis of Cushing’s disease can be challenging. The best imaging modality, MRI, may be normal in up to 40% of patients (Swearingen et al. 2004). Inferior petrosal sinus sampling (IPSS) is the gold standard for differentiating between pituitary and non-pituitary sources of corticotrophin, with diagnostic accuracy of 87% (Swearingen et al. 2004) but is a highly skilled and invasive technique, requiring placement of catheters in both inferior petrosal sinuses (Zhao et al. 2014). [68Ga]Ga-DOTA-TATE is also useful in localization of ectopic ACTH producing tumors. Ectopic ACTH secretion is an infrequent cause of ACTH-dependent Cushing’s syndrome. It often presents a major diagnostic difficulty because it is hard to differentiate Cushing’s disease from ectopic tumors and is often difficult to localize. [68Ga]Ga-DOTA-TATE could detect ectopic ACTH source in 5/6 patients presenting de novo with ACTH-dependent Cushing’s and 1/2 patients with treated pituitary Cushing’s. Three with ectopic Cushing’s had lung carcinoid. Although pulmonary carcinoids showed positive uptake of [68Ga]Ga-DOTA-TATE, the level of accumulation was unusually low (SUVmax 1.4–2.0).

There are varying results regarding use of conventional single photon somatostatin receptor scintigraphy (SRS) for evaluating patients with ectopic Cushing syndrome with majority of published studies (Tabarin et al. 1999; Tsagarakis et al. 2003; Özkan et al. 2013; Ejaz et al. 2011; Torpy et al. 1999; Zemskova et al. 2010; Ilias et al. 2005; Isidori et al. 2006; Doi et al. 2010; Kakade et al. 2013; Gilardi et al. 2014) reporting sensitivity of between 40 and 60%. In the two largest published studies by Zemskova et al. and Ilias et al. SRS could detect ectopic tumor in (17/30) 57% and (21/43) 49% (Zemskova et al. 2010; Ilias et al. 2005). Studies comparing SRS with diagnostic CT/MRI have found limited role for SRS as no additional lesions were detected with SRS relative to CT/MRI (Haug et al. 2010; Özkan et al. 2013; Ejaz et al. 2011).

Studies evaluating [68Ga]Ga-DOTA-TATE-labeled somatostatin ligands are limited to a small number of case reports and very small case series (Özkan et al. 2013; Kakade et al. 2013; Gilardi et al. 2014; Venkitaraman et al. 2014; Treglia et al. 2013; Därr et al. 2012; Thomas et al. 2013; Willhauck et al. 2012; Schalin-Jäntti et al. 2012; Gani et al. 2011). The sensitivity of [68Ga]Ga-DOTA-TATE in detecting source of ectopic ACTH secretion from previously published cases is approximately 72% (18/25 patients) (Table 3). Ozkan et al. (Özkan et al. 2013) found positive Ga-68-DOTATATE uptake in only 2/5 patients with ectopic ACTH syndrome; one patient showed false positive uptake. In contrast, Gilardi et al. reported that [68Ga]Ga-DOTA-TATE revealed the source of ectopic lesions in 5/5 patients with ectopic ACTH syndrome (Gilardi et al. 2014). In 3/5 patients, SRS with [111In]In-pentetreotide had failed to localize the source of ACTH secretion. Kakade et al. reported that [68Ga]Ga-DOTA-TATE was positive in 4/6 ectopic ACTH secreting tumor including two which had negative CT (Kakade et al. 2013). In our series, 6 of 8 ectopic ACTH secreting tumors were detected with [68Ga]Ga-DOTA-TATE; in one case, ectopic primary tumor was not seen on CT but was depicted with [68Ga]Ga-DOTA-TATE.

**Table 3:** Study of ectopic ACTH PET-CT scan with ^68^Ga-DOTA peptides

Studies	Patients	Positive uptake within ectopic tumor	Negative uptake within ectopic tumor	Tracer	Tumor types
Veit et al. (2013)	1	1	0	^68^Ga-DOTANOC	Paranasal adenoma
Singer et al. (2010)	1	1	0	^68^Ga-DOTATOC	Ileum carcinoma
Ozkan et al. (2013)	5	2	3*	^68^Ga-DOTATATE	Bronchial carcinoid-1, metastatic atypical carcinoid-1
Kakade et al. (2013)	6	4	2	^68^Ga-DOTATATE	Bronchial carcinoid-1, PNET-1, MTC-2
Gilardi et al. (2014)	5	5	0	^68^Ga-DOTATOC	Bronchial carcinoid
Venkitaraman et al. (2014)	3	3	0	^68^Ga-DOTATOC	Bronchial carcinoid
Treglia et al. (2013)	1	1	0	^68^Ga-DOTANOC	PNET
Darr et al. (2012)	1	1	0	^68^Ga-DOTATATE	Bronchial carcinoid
Thomas et al. (2013)	1	1	0	^68^Ga-DOTATATE	Nasal paraganglioma
Willhauck et al. (2012)	1	1	0	^68^Ga-DOTATATE	Sphenoid adenoma
Schalin-Jäntti et al. (2012)	1	0	1	^68^Ga-DOTATOC	Bronchial carcinoid
Gani et al. (2011)	1	0	1	^68^Ga-DOTATOC	Bronchial carcinoid
Our study	8	6	2	^68^Ga-DOTATATE	Bronchial carcinoid-3, ileum carcinoma-1, PNET-2

*MTC* medullar thyroid carcinoma, *PNET* pancreatic neuroendocrine tumor

*One false positive

Interestingly, pituitary uptake seen in patients with recurrent pituitary adenomas was less intense than pituitary uptake in patients with de novo ectopic Cushing’s syndrome (mean SUVmax 4.1 vs 6.2 respectively). The results are in line with the literature data, although absolute values of SUV are generally lower in our case (Kakade et al. 2013; Gilardi et al. 2014; Venkitaraman et al. 2014; Treglia et al. 2013; Därr et al. 2012; Thomas et al. 2013; Willhauck et al. 2012; Schalin-Jäntti et al. 2012; Gani et al. 2011).

Thyrotropinomas are a rare cause of hyperthyroidism in clinical practice often diagnosed as macro adenomas due to delayed diagnosis. Suppression of TSH secretion is mediated via both SSTR 2 and SSTR 5 subtypes (Shimon et al. 1997). Long acting somatostatin analog drugs reduce TSH secretion and normalize FT4 and FT3 levels in 90% of patients suffering with pituitary TSH secreting tumors (Ben-Shlomo and Melmed 2010). In 25% of thyrotropinoma cases, there is autonomous secretion of a second pituitary hormone (Elhadd et al. 2009). One of our 9 patients with recurrent Cushing’s syndrome also had the symptoms of central hyperthyroidism with final diagnosis of plurihormonal pituitary adenoma with expression of ACTH, FSH, and TSH (case 7, Table 2). In a study published by Foppiani et al. all 3 patients with TSH-oma were positive in octreoscan (Foppiani et al. 2007). Despite previous pituitary surgery, there was moderate increased [68Ga]Ga-DOTA-TATE uptake in patient with recurrent Cushing’s and thyrotropinoma and intense uptake in 3 cases of recurrent thyrotropinomas (Fig. 4). Moderate to high pituitary uptake was also seen in both patients with recurrent prolactinomas.

## Conclusion

[68Ga]Ga-DOTA-TATE, with integrated PET/CT, is a useful diagnostic modality for the evaluation of patients with suspected pituitary pathology. Recurrent Cushing’s disease is associated with positive pituitary uptake of [68Ga]Ga-DOTA-TATE. Although in these cases it would not be possible to distinguish pathological from physiological uptake, positive [68Ga]Ga-DOTA-TATE is useful as it indicates the presence of functioning pituitary tissue. Absence of pituitary uptake in patients with recurrent Cushing’s suggests source of ACTH is ectopic. Moderate to high pituitary tracer uptake of [68Ga]Ga-DOTA-TATE was seen in patients with recurrent thyrotropinomas and prolactinomas indicating [68Ga]Ga-DOTA-TATE may be useful for detection of disease post-surgery.

[68Ga]Ga-DOTA-TATE may be helpful in detecting source of ectopic lesion in Cushing’s syndrome particularly in those where CT imaging is negative. Finally, locally aggressive or metastatic pituitary tumors may show [68Ga]Ga-DOTA-TATE uptake and therefore indicate potential for treatment with radio-labeled somatostatin receptor analogues such as[177Lu]Lu DOTA-TATE.

## Data Availability

All data and materials are available by the authors.
